# Swipe-based dating applications use and its association with mental health outcomes: a cross-sectional study

**DOI:** 10.1186/s40359-020-0373-1

**Published:** 2020-03-04

**Authors:** Nicol Holtzhausen, Keersten Fitzgerald, Ishaan Thakur, Jack Ashley, Margaret Rolfe, Sabrina Winona Pit

**Affiliations:** 1 Western Sydney University, University Centre for Rural Health, 61 Uralba Street, Lismore, NSW 2480 Australia; 2 University Of Sydney, School of Medicine, Sydney, Australia

## Abstract

**Background:**

Swipe-Based Dating Applications (SBDAs) function similarly to other social media and online dating platforms but have the unique feature of “swiping” the screen to either like or dislike another user’s profile. There is a lack of research into the relationship between SBDAs and mental health outcomes.

The aim of this study was to study whether adult SBDA users report higher levels of psychological distress, anxiety, depression, and lower self-esteem, compared to people who do not use SBDAs.

**Methods:**

A cross-sectional online survey was completed by 437 participants. Mental health (MH) outcomes included the Kessler Psychological Distress Scale, Generalised Anxiety Disorder-2 scale, Patient Health Questionnaire-2, and Rosenberg Self-Esteem Scale. Logistic regressions were used to estimate odds ratios of having a MH condition. A repeated measures analysis of variance was used with an apriori model which considered all four mental health scores together in a single analysis. The apriori model included user status, age and gender.

**Results:**

Thirty percent were current SBDA users. The majority of users and past users had met people face-to-face, with 26.1%(60/230) having met > 5 people, and only 22.6%(52/230) having never arranged a meeting. Almost 40%(39.1%; 90/230) had previously entered into a serious relationship with someone they had met on a SBDA. More participants reported a positive impact on self-esteem as a result of SBDA use (40.4%; 93/230), than a negative impact (28.7%;66/230).

Being a SBDA user was significantly associated with having psychological distress (OR = 2.51,95%CI (1.32–4.77)), *p* = 0.001), and depression (OR = 1.91,95%CI (1.04–3.52), *p* = 0.037) in the multivariable logistic regression models, adjusting for age, gender and sexual orientation. When the four MH scores were analysed together there was a significant difference (*p* = 0.037) between being a user or non-user, with SDBA users having significantly higher mean scores for distress (*p* = 0.001), anxiety (*p* = 0.015) and depression (*p* = 0.005). Increased frequency of use and longer duration of use were both associated with greater psychological distress and depression (*p* < 0.05).

**Conclusion:**

SBDA use is common and users report higher levels of depression, anxiety and distress compared to those who do not use the applications. Further studies are needed to determine causality and investigate specific patterns of SBDA use that are detrimental to mental health.

## Background

Swipe-Based Dating Applications (SBDAs) provide a platform for individuals to interact and form romantic or sexual connections before meeting face-to-face. SBDAs differ from other online dating platforms based on the feature of swiping on a mobile screen. Each user has a profile which other users can approve or reject by swiping the screen to the right or the left. If two individuals approve of each other’s profiles, it is considered a “match” and they can initiate a messaging interaction. Other differentiating characteristics include brief, image-dominated profiles and the incorporation of geolocation, facilitating user matches within a set geographical radius. There are a variety of SBDAs which follow this concept, such as Tinder, Bumble, Happn, and OkCupid.

The Australian population of SBDA users is rapidly growing. In 2018, Tinder was the most popular mobile dating app in Australia, with approximately 57 million users worldwide [1, 2]. Most SBDA users are aged between 18 and 34, and the largest increase in SBDA use has been amongst 18–24 year-olds. However, there has also been a sharp increase in SBDA use amongst 45–54 year-olds, rising by over 60%, and 55–64 year-olds, where SBDA use has doubled [3]. SBDA use is also rising internationally; of internet users in the United States, 19% are engaging in online dating (sites or applications) [4]. The role of SBDAs in formation of long term relationships is already significant and also rising; a 2017 survey of 14,000 recently married or engaged individuals in the United States found that almost one in five had met their partner via online dating [5]. A large, nationally representative survey and audit conducted by eHarmony predicted that by 2040, 70% of relationships will begin online [6].

With SBDA use increasing at such a rapid rate, investigation into the health implications of these applications is warranted. Such research has to date focused on investigating the link between these applications and high-risk sexual behaviour, particularly in men who have sex with men [7]. Currently, there is a paucity of research into the health impacts of SBDAs, especially with regards to mental health [8].

The significance of mental health as a public health issue is well established [9, 10]; of Australians aged 16–85, 45% report having experienced a mental illness at least once in their lifetime. Amongst 18–34 year-olds, those who use SBDAs most, the annual prevalence of mental illness is approximately 25% [11]. Moreover, mental illness and substance abuse disorders were estimated to account for 12% of the total burden of disease in Australia [10]. However, mental health refers not only to the absence of mental illness, but to a state of wellbeing, characterised by productivity, appropriate coping and social contribution [12]. Therefore, while mental illness presents a significant public health burden and must be considered when investigating the health impacts of social and lifestyle factors, such as SBDA use, a broader view of implications for psychological wellbeing must also be considered.

A few studies have investigated the psychological impact of dating applications, assessing the relationship between Tinder use, self-esteem, body image and weight management. Strubel & Petrie found that Tinder use was significantly associated with decreased face and body satisfaction, more appearance comparisons and greater body shame, and, amongst males, lower self-esteem [8]. On the other hand, Rönnestad found only a weak relationship between increased intensity of Tinder use and decreased self-esteem; however this may be explained by the low intensity of use in this study. Correlations were 0.18 or lower for self-esteem and the scores for app usage, dating behaviour and tinder intensity [13]. A study by Tran et al. of almost 1800 adults found that dating application users were significantly more likely to engage in unhealthy weight control behaviours (such as laxative use, self-induced vomiting and use of anabolic steroids) compared to non-users [14].

To our knowledge, there have been no studies investigating the association between SBDA use and mood-based mental health outcomes, such as psychological distress or features of anxiety and depression. However, there have been studies investigating the relationship between mental health outcomes and social media use. SBDAs are innately similar to social media as they provide users a medium through which to interact and to bestow and receive peer approval; the ‘likes’ of Facebook and Instagram are replaced with ‘right swipes’ on Tinder and Bumble [8].

To date, research into the psychological impact of social media has yielded conflicting evidence. One study found a significant, dose-response association of increased frequency of social media use (with measures such as time per day and site visits per week) with increased likelihood of depression [15]. Contrarily, Primack et al. found the use of multiple social media platforms to be associated with symptoms of depression and anxiety independent of the total amount of time spent of social media [16]. However, some studies found no association between social media use and poorer mental health outcomes, such as suicidal ideation [17–19]. Other studies have investigated other aspects of use, beyond frequency and intensity; ‘problematic’ Facebook use, defined as Facebook use with addictive components similar to gambling addiction, has been associated with increased depressive symptoms and psychological distress [20, 21]. A study of 18–29 year olds by Stapleton et al. found that while Instagram use did not directly impact user self-esteem, engaging in social comparison and validation-seeking via Instagram did negatively impact self-esteem [22]. A meta-analysis by Yoon et al. found a significant association between total time spent on social media and frequency of use with higher levels of depression [23]. This analysis also found that social comparisons made on social media had a greater relationship with depression levels than the overall level of use [23], providing a possible mediator of effect of social media on mental health, and one that may be present in SBDAs as well.

Existing research on the connection between social media use and mental health outcomes suggests that the way these applications and websites are used (to compare [22, 23]; to seek validation [22]; with additive components [20, 21]) is more significant than the frequency or time spent doing so. This validation-seeking is also seen in SBDAs.

Strubel & Petrie argue that SBDAs create a paradigm of instant gratification or rejection, placing users in a vulnerable position [8]. Furthermore, Sumter et al. found the pursuit of self-worth validation to be a key motivation for Tinder use in adults, further increasing the vulnerability of users to others’ acceptance or rejection [24]. This, combined with the emphasis placed on user images in SBDA [25], enhances the sexual objectification in these applications. The objectification theory suggests that such sexual objectification leads to internalisation of cultural standards of attractiveness and self-objectification, which in turn promotes body shame and prevents motivational states crucial to psychological wellbeing [8, 26]. The pursuit of external peer validation seen in both social media and SBDAs, which may be implicated in poorer mental health outcomes associated with social media use, may also lead to poorer mental health in SBDA users.

This study aimed to investigate the relationship between Swipe-Based Dating Applications (SBDAs) and mental health outcomes by examining whether SBDA users over the age of 18 report higher levels of psychological distress, anxiety, depression, and lower self-esteem, compared to people who do not use SBDAs. Based on the similarities between social media and SBDAs, particularly the exposure to peer validation and rejection, we hypothesised that there would be similarities between the mental health implications of their use. As the pursuit of validation has already been found to be a motivator in Tinder use [24], and implicated in the adverse mental health impacts of social media [22], we hypothesised that SBDA users would experience poorer mental health compared to people who did not use SBDAs, reflected in increased psychological distress, symptoms of anxiety and depression, and lower self-esteem.

## Methods

### Recruitment and data collection

A cross sectional survey was conducted online using convenience sampling over a 3 month period between August and October 2018. Participants were recruited largely online via social media, including Facebook and Instagram. Administrative approval was sought before posting the survey link in relevant groups on these sites, including dating groups such as “Facebook Dating Australia” and community groups. A link to the survey was also disseminated by academic organisations and the Positive Adolescent Sexual Health Consortium. The survey was also disseminated via personal social networks, such as personal social media pages. The survey was created online using the secure Qualtrics software (version Aug-Oct 2018 Qualtrics, Provo, Utah).

### Measures

Demographic factors, dating application factors and mental health outcomes were measured. Demographic measures included age, gender, sexual orientation, relationship/marital status, employment status and use of other social media platforms. The questionnaire also included basic information on SBDA usage. Initially respondents were asked if they were current users, past users or non-users. Past users were those who had not used an SBDA in the last 6 months. This variable was dichotomised into “current users” (used an SBDA within the last 6 months) and “non-users” (have never used or have not used an SBDA in the last 6 months). The survey included frequency of SBDA use and duration of use. Respondents were also asked the number of people they met in person from SBDAs, the number of serious relationships with people they met on SBDAs and if they met their current partner on an SBDA. Self-reported impact of SBDAs on self-esteem was assessed using a five-point scale from very negatively to very positively. Due to small numbers in the extreme categories this variable was simplified to positively, no impact and negatively. Past users and non-users were asked their reason for not using SBDAs and what other methods they used to meet potential partners.

The outcome measures included psychological distress, anxiety, depression, and self-esteem. In line with the Australian Bureau of Statistics [27], psychological distress was assessed using the Kessler Psychological Distress Scale (K6). The K6 has six questions asking the frequency of various symptoms, each with a score of 0–4 (none, a little, some, most or all of the time). The total score is out of 24, with scores over 13 indicating distress. Validity was assessed and confirmed by using data from 14 countries and recommended that it can be used when brief measures are required [28].

Anxiety was measured using the Generalised Anxiety Disorder-2 scale (GAD-2). This scale involves two questions asking how many days they have experienced symptoms of anxiety in the last 2 weeks. Each question is scored from 0 to 3 (not at all, several days, more than half the days, nearly everyday), resulting in a total out of six. A systematic review and diagnostic meta-analysis of the international literature demonstrated that scores greater than or equal to three indicated anxiety [27]. Construct validity of the GAD-2 was confirmed by intercorrelations with demographic risk factors for depression and anxiety and other self-report scales in a German population [29].

Depression was measured using the Patient Health Questionnaire-2 (PHQ-2), which has two questions asking how many days in the last 2 weeks they have experienced low mood or anhedonia. The scoring system is the same as the GAD-2. Construct validity of the PHQ-2 was confirmed by intercorrelations with demographic risk factors for depression and anxiety and other self-report measures in a German population [29]. The PHQ-2 threshold of ≥3 was also the best balance between sensitivity (91%) and specificity (78%) for detecting possible cases of depression in a sample of 3626 Australian general practice patients [30].

Finally, self-esteem was measured using the Rosenberg Self-Esteem Scale (RSES). This scale has ten statements related to self-esteem and respondents are required to “strongly agree”, “agree”, “disagree” or “strongly disagree” with each one. An example statement is: “At times I think I am no good at all”. Some of the statements are inversely scored, in order for low scores (< 15/30) to indicate low self esteem [31].

All of these tools (K6, GAD-2, PHQ-2, RSES) are widely used and have demonstrated validity. The cut off scores were used to dichotomise the variables to assess for the presence of the particular mental health outcome (psychological distress, anxiety, depression or low self-esteem). The cut off scores were provided by the relevant literature for each tool [27–29, 31].

### Statistical analysis

Descriptive statistics were calculated, using SPSS software V22 (IBM, New York, USA), to describe the sample and outcome measures. Chi-square and Fisher’s exact were used to determine the initial association between the independent factors and the four dependent mental health variables. Significance level was set at a *p* < 0.05. A cronbach’s alpha analysis was conducted on the items within each of the four mental health scales to assess the level of internal consistency.

The mental health (MH) outcomes were considered in two ways. Firstly, MH outcomes were considered as binary outcomes of not having or having psychological distress, anxiety, depression, or normal or low for self-esteem using univariate and multivariate logistic regression. Secondly, the continuous scores for each of the MH outcomes were compared with using apps versus not using apps using profile analysis with a repeated measures analysis of variance (RM ANOVA). Profile analysis was chosen because it is commonly used when there are various measures of the same dependent variable. Profile analysis is the “multivariate equivalent of repeated measures or mixed ANOVA” [6].

Univariable logistic regressions were used to estimate crude odds ratios to determine which factors are associated with having poorer mental health. For the multivariable logistic regression, the mental health outcome measures were the dependent variable and user status was the variable of interest whilst being adjusted for age, gender and sexual orientation.

The profile analysis considers mean levels of the four continuous MH outcomes (within-subject factors) together in the one analysis and provides an adjustment for the lack of independence of these measures. This analysis was conducted to provide a different picture to that of simply measuring whether someone has a specific MH condition as the numbers were rather small. User status was the variable of interest. Age and gender were included in the apriori model for adjustment. This analysis provides an understanding of how user status is related to the magnitude of MH scores after adjusting for gender and age (between-subject factors). The self-esteem outcome was reversed (30 minus score) so that higher scores were indicative of worse MH outcomes. Both the Wilks lambda and Greenhouse-Geiser results are presented as the sphericity assumption was not met.

### Ethics

Ethics approval was granted by Western Sydney University Human Research Ethics Committee (H11327).

## Results

### Sample

Five-hundred-and-twenty people completed the online survey. After excluding those under the age of 18 and those who resided outside of Australia, 475 valid responses remained. The final sample consisted of 437 respondents who answered the “user status” question.

### Sample characteristics

One in three of the total 437 participants were using a dating app (29.5%, *n* = 129), 23.1% (*n* = 101) were past users and 47.4% (*n* = 207) had never used a dating app. Our sample had a high proportion of people aged 18–23 (53.6%, *n* = 234), females (58.4%, *n* = 253) and lesbian, gay, bisexual, transgender, queer, intersex, plus (LGBTQI+) individuals (13.3%, *n* = 58) (Table 1). The majority of participants were in an exclusive relationship (53.5%, *n* = 231). Of the participants, 23.4% (*n* = 102) were unemployed and 100% (*n* = 434) used social media at least once per week.

**Table 1:** Demographics (*n* = 437)

	Total n (%)		Non-users n (%)	Past Users n (%)	Users n (%)	Chi-square value	Degrees of Freedom	*P*-value^a^
n %	437		207 (47.4)	101 (23.1)	129 (29.5)			
Age* (missing = 1)								
18–23	234	53.6	91 (38.9)	56 (23.9)	87 (37.2)	18.949	4	**0.001**
24–29	99	22.8	52 (52.5)	24 (24.2)	23 (23.2)			
30 and older	103	23.7	63 (61.2)	21 (20.4)	19 (18.4)			
Gender (missing = 4)								
Male	180	41.6	80 (44.4)	45 (25.0)	55 (30.6)	1.222	2	0.543
Female	253	58.4	125 (49.4)	54 (21.3)	74 (29.2)			
Sexual orientation								
Heterosexual	379	86.7	193 (50.9)	84 (22.2)	102 (26.9)	15.303	2	**< 0.001**
LGBTQI+	58	13.3	14 (24.1)	17 (29.3)	27 (46.6)			
Marital status (missing = 1)								
Married/de facto	99	22.7	78 (78.8)	19 (19.2)	2 (2.0)	59.926	2	**< 0.001**
Not married	337	77.3	129 (38.3)	82 (24.3)	126 (37.4)			
Relationship status (missing = 5)								
Single & not dating	141	32.6	58 (41.1)	16 (11.3)	67 (47.5)	160.562	4	**< 0.001**
Dating	60	13.9	6 (10.0)	6 (10.0)	48 (80.0)			
In an exclusive relationship	231	53.5	138 (59.7)	79 (34.2)	14 (6.1)			
Employment (missing = 1)								
Not employed	102	23.4	48 (47.1)	21 (20.6)	33 (32.4)	2.952	4	0.566
0–30 h per week	170	39.0	80 (47.1)	36 (21.2)	54 (31.8)			
> 30 h per week	164	37.6	78 (47.6)	44 (26.8)	42 (25.6)			
Social media use (missing = 3)								
≥ once a week	434	100	129 (29.7)	101 (23.3)	204 (47.0)	–	–	–

^a^ Chi-square analyses

*p* value for a significant result

### Demographics and user status

While 37.2% (*n* = 87) of those aged 18–23 were users, only 18.4% (*n* = 19) of those aged 30 or older had used an app in the last 6 months (Table 1). A statistically significant higher proportion of LGBTQI+ participants (46.6%; *n* = 27) used SBDAs compared to heterosexuals (26.9%; *n* = 102) (*p* < 0.001). Participants that were dating were significantly more likely to use SBDAs (80%, *n* = 48) than those who were not dating (47.5%, *n* = 67) or were in an exclusive relationship (6.1%, *n* = 14) (*p* < 0.001). There was no significant difference in user status based on gender or employment status.

### Patterns of use and non-use

Table 2 displays characteristics of dating app use in our sample. The most-used SBDA was Tinder, with 30% of our total sample, and 100% of current users, using the app. Bumble was also widely-used, however had less than half the number of users that Tinder did (*n* = 61; 47.3%). Among SBDA users, the majority (51.2%; *n* = 66) had been using SBDAs for over a year.

**Table 2:** Patterns of App Use and Non-use (*N* = 437)

Characteristics	N(%)
Users (*n* = 129)	
Frequency of SBDA Use	Frequency n (%)
Less than once a week	40 (31.0%)
Once a week or more but less than daily	55 (42.6%)
Daily	34 (26.4%)
Duration of Use (missing = 1)	
≤ 12 months	62 (48.1%)
More than a year	66 (51.2%)
Most-Used SBDAs	
Tinder	129 (100%)
Bumble	61 (47.3%)
Plenty Of Fish	10 (7.8%)
Grindr	8 (6.2%)
Coffee Meets Bagel	8 (6.2%)
Users & Past Users (*n* = 230)	
Number of people met face-to-face	Frequency n (%)
0	52 (22.6%)
1–2	65 (28.3%)
3–5	53 (23.0%)
> 5	60 (26.1%)
Number of serious relationships	
None	140 (60.9%)
One or more	90 (39.1%)
How do you feel that the use of dating apps has impacted your self-esteem?	
Positive	93 (40.4%)
No impact	71 (30.0%)
Negative	66 (28.7%)
Non-Users & Past Users (*n* = 308)	
Reasons for not using or no longer using dating applications (missing = 8)	Frequency n (%)
Not looking for a relationship	201 (67.0%)
Prefer to meet people in other ways	94 (31.3%)
Don’t trust people to be honest online	33 (11.0%)
Don’t cater for the kind of relationship I want	30 (10.0%)
Negative social stigma	20 (6.7%)
Other	9 (3.0%)
I don’t think I will match with anyone	12 (4.0%)
Previous bad experience	1 (0.3%)
Where people meet past partners other than SBDA (missing = 8)	
Through work/university/school	146 (48.7%)
Through mutual friends	112 (37.3%)
Through church or hobbies	37 (12.3%)
At bars/clubs or other social venues	30 (10.0%)
Other	30 (10.0%)
At parks/libraries/other public places	6 (2.0%)

The majority of users and past users had met people face-to-face, with 26.1% (*n* = 60) having met over five people, and only 22.6% (*n* = 52) having never arranged a meeting. Almost 40% (39.1%; *n* = 90) of current or past users had previously entered into a serious relationship with someone they had met on a SBDA. More participants reported a positive impact on self-esteem as a result of SBDA use (40.4%; *n* = 93), than a negative impact (28.7%; *n* = 66).

Among those who did not use SBDAs, the most common reason for this was that they were not looking for a relationship (67%; *n* = 201), followed by a preference for meeting people in other ways (31.3%; 94/300), a mistrust of people online (11%; 33/300) and feeling that these applications do not cater for the kind of relationship they were seeking (10%; 30/300). Non-users had most often met past partners through work, university or school (48.7%; 146/300) or through mutual friends (37.3%; 112/300).

### Reliability analysis

All four mental health scales demonstrated high levels of internal consistency. The Cronbach’s alpha was 0.865 for K6, 0.818 for GAD-2, 0.748 for PHQ-2 and 0.894 for RSES.

### SBDA use and mental health outcomes

A statistically significant association from chi-square analyses was demonstrated between psychological distress and user status (*P* < 0.001), as well as depression and user status (*P* = 0.004) (Table 3). While a higher proportion of users met the criteria for anxiety (24.2%; 31/128) and poor self-esteem (16.4%; 21/128), this association was not statistically significant.

**Table 3:** Current dating app users versus non-users by mental health outcome (*N* = 437)

MH Measure		Total n %		Non-Users	Users	Chi-square value	Degrees of Freedom	*P* value^a^
K6 Score - Psychological Distress	No distress	388	88.8%	285 (92.5%)	103 (79.8%)	12.701	1	**< 0.001**
	Distress	49	11.2%	23 (7.5%)	26 (20.2%)			
GAD-2 Score – Anxiety (missing = 1)	No Anxiety	345	79.1%	248 (80.5%)	97 (75.8%)	1.229	1	0.268
	Anxiety	91	20.9%	60 (19.5%)	31 (24.2%)			
PHQ-2 Score – Depression	No Depression	383	87.6%	279 (90.6%)	104 (80.6%)	8.335	1	**0.004**
	Depression	54	12.4%	29 (9.4%)	25 (19.4%)			
Rosenberg Self-Esteem Scale (missing = 7)	Normal/High	369	85.8%	262 (86.8%)	107 (83.6%)	0.738	1	0.390
	Low	61	14.2%	40 (13.2%)	21 (16.4%)			

^a^ Chi-square analyses

*p* value for a significant result

### Univariate logistic regression

Univariate logistic regression demonstrated a statistically significant relationship between age and all four mental health outcomes, with younger age being associated with poorer mental health (*p* < 0.05 for all). Female gender was also significantly associated with anxiety, depression, and self-esteem (*p* < 0.05) but not distress. Sexual orientation was also significant, with LGBTQI+ being associated with higher rates of all mental health outcomes (p < 0.05). Being in an exclusive relationship was associated with lower rates of psychological distress (*p* = 0.002) and higher self-esteem (*p* = 0.018).

Users had three times the odds of being psychologically distressed than non-users (OR: 3.13, 95%CI 1.71–5.73, *p* < 0.001) and twice the odds of being depressed (OR 2.31, 95% CI 1.29–4.13, *p* = 0.005). Increased frequency of use was associated with increased risk of psychological distress and depression. People who used SBDAs daily were almost four times more likely to be distressed (OR: 3.79, 95% CI 1.54–9.30, *p* = 0.004) or depressed (OR: 3.98, 95% CI 1.73–9.14, *p* = 0.001) when compared to those who never use. Those who had used SBDAs for over a year, had three and half times the odds of being psychologically distressed than non-users (OR: 3.55, 95% CI 1.74–7.25, *p* = 0.001) and three times the odds of being depressed (OR: 3.00, 95% CI 1.52–5.91, *p* = 0.002). Number of serious relationships and self-reported impact on self-esteem were not associated with any of the four outcome variables Table 4.

**Table 4:** Association between independent variables and binary mental health outcomes – univariate analyses (*N* = 437)^a^

		Psychological Distress Crude OR (95% CI)	*P*-value	Anxiety Crude OR (95% CI)	*P*-value	Depression Crude OR (95% CI)	*P*-value	Self-Esteem Crude OR (95% CI)	*P*-value
Demographics	Age	*N* = 436		*N* = 435		*N* = 436		*N* = 429	
		0.90 (0.84–0.97)	0.003	0.95 (0.92–0.98)	0.002	0.96 (0.92–0.99)	0.022	0.93 2(0.82–0.98)	**0.003**
	Age	*N* = 436	0.005	*N* = 435	0.010	*N* = 436	0.129	*N* = 429	**0.028**
	18–23	6.26 (1.89–20.81)	0.003	2.69 (1.38–5.25)	0.004	1.90 (0.88–4.10)	0.103	3.35 (1.37–8.16)	**0.008**
	24–29	3.33 (0.88–12.70)	0.078	1.69 (0.77–3.71)	0.195	1.04 (0.40–2.75)	0.930	2.55 (0.94–6.94)	0.066
	30+	REF		REF		REF		REF	
	Gender	*N* = 433		*N* = 432		*N* = 433		*N* = 426	
	Male	REF		REF		REF		REF	
	Female	1.84 (0.96–3.54)	0.068	2.02 (1.22–3.34)	0.006	2.23 (1.17–4.23)	0.015	2.01 (1.10–3.65)	**0.022**
	Sexual Orientation	*N* = 437		*N* = 436		*N* = 437		*N* = 430	
	Heterosexual	REF		REF		REF		REF	
	LGBTQI+	3.54 (1.78–7.02)	< 0.001	2.28 (1.25–4.15)	0.007	2.70 (1.36–5.35)	0.005	2.11 (1.06–4.21)	**0.035**
	Relationship Status	*N* = 432	0.004	*N* = 431	0.541	*N* = 432	0.136	*N* = 426	0.055
	Single & not dating	REF		REF		REF		REF	
	Dating	0.98 (0.43–2.19)	0.951	0.65 (0.30–1.42)	0.278	1.08 (0.48–2.45)	0.850	0.85 (0.38–1.89)	0.683
	In an Exclusive relationship	0.34 (0.17–0.67)	0.002	0.85 (0.51–1.41)	0.528	0.57 (0.30–1.07)	0.081	0.49 (0.27–0.89)	**0.018**
	Employment	*N* = 436	0.100	*N* = 435	0.944	*N* = 436	0.435	*N* = 429	0.818
	Not employed	2.36 (1.07–5.21)	0.034	1.11 (0.60–2.03)	0.746	1.47 (0.69–3.16)	0.322	1.26 (0.62–2.54)	0.527
	0–30 h per week	1.79 (0.85–3.76)	0.127	1.07 (0.63–1.81)	0.812	1.52 (0.78–2.98)	0.222	1.12 (0.60–2.10)	0.727
	> 30 h per week	REF		REF		REF		REF	
SBDA User Status	User Status	*N* = 437		*N* = 436		*N* = 437		*N* = 430	
	Non-User	REF		REF		REF		REF	
	User	3.13 (1.71–5.73)	< 0.001	1.32 (0.81–2.16)	0.268	2.31 (1.29–4.13)	0.005	1.29 (0.72–2.28)	0.391
SBDA Use	Frequency of Use	*N* = 437	0.003	*N* = 436	0.125	*N* = 437	0.010	*N* = 430	0.369
	Never	REF		REF		REF		REF	
	Less than once a week	2.61 (1.04–6.55)	0.041	2.30 (1.23–4.68)	0.022	1.69 (0.65–4.35)	0.281	0.77 (0.26–2.27)	0.629
	Once a week or more	2.94 (1.35–6.44)	0.007	0.87 (0.42–1.83)	0.717	1.79 (0.80–4.12)	0.158	1.60 (0.76–3.35)	0.212
	Daily	3.79 (1.54–9.30)	0.004	1.06 (0.44–2.56)	0.892	3.98 (1.73–9.14)	0.001	1.74 (0.71–4.25)	0.228
	Duration of Use	*N* = 436	0.001	*N* = 435	0.544	*N* = 436	0.006	*N* = 429	0.510
	Never	REF		REF		REF		REF	
	≤ 12 months	2.60 (1.20–5.66)	0.016	1.20 (0.62–2.31)	0.595	1.59 (0.71–3.55)	0.256	1.26 (0.59–2.69)	0.545
	More than a year	3.55 (1.74–7.25)	0.001	1.39 (0.75–2.59)	0.292	3.00 (1.52–5.91)	0.002	1.49 (0.73–3.03)	0.273
	Number of people met face-to-face	*N* = 232	0.340	*N* = 231	0.628	*N* = 232	0.246	*N* = 229	0.129
	0	REF		REF		REF		REF	
	1–2	2.23 (0.66–7.56)	0.199	1.74 (0.73–4.15)	0.216	2.77 (0.84–9.18)	0.095	3.36 (1.04–10.93)	**0.044**
	3–5	2.13 (0.60–7.56)	0.242	1.47 (0.59–3.69)	0.412	2.45 (0.71–8.51)	0.158	2.23 (0.63–7.91)	0.216
	> 5	3.06 (0.92–10.16)	0.067	1.66 (0.68–4.04)	0.264	3.39 (1.03–11.14)	0.044	3.81 (1.17–12.44)	**0.027**
	Number of serious relationships	*N* = 232		*N* = 231		*N* = 232		*N* = 229	
	None	REF		REF		REF		REF	
	One or more	1.12 (0.54–2.36)	0.758	1.06 (0.58–1.94)	0.848	1.34 (0.67–2.71)	0.412	1.33 (0.67–2.65)	0.418
	Self-reported impact on self esteem	*N* = 232	0.391	*N* = 231	0.864	*N* = 232	0.428	*N* = 229	0.556
	Positive	1.26 (0.49–3.24)	0.625	1.22 (0.60–2.47)	0.591	1.31 (0.54–3.19)	0.555	0.85 (0.36–1.96)	0.697
	No impact	REF		REF		REF		REF	
	Negative	1.90 (0.73–4.92)	0.188	1.10 (0.51–2.40)	0.806	1.82 (0.73–4.54)	0.199	1.33 (0.56–3.13)	0.518

^a^ *OR* Odds ratio, *REF* reference category

*p* value for a significant result

### Multivariate logistic regression

After adjusting for age, gender and sexual orientation in a multivariate model, user status was still significantly associated with distress and depression, but not anxiety and self-esteem, (Table 5). Users had 2.5 times the odds of being psychologically distressed than non-users (OR: 2.51, 95% CI 1.32–4.77, *p* = 0.005) and almost twice the odds of being depressed (OR: 1.91, 95% CI 1.04–3.52, *p* = 0.037).

**Table 5:** Multivariate logistic regression results for user status with the binary mental health outcomes (*N* = 437)^1^

	Psychological Distress AOR (95% CI)*	*P*-value	Anxiety AOR (95% CI)	*P*-value	Depression AOR (95% CI)	*P* value	Self-Esteem AOR (95% CI)	*P*-value
User Status	*N* = 432		*N* = 431		*N* = 432		*N* = 425	
Non-User	REF		REF		REF		REF	
User	2.51 (1.32–4.77)	**0.005**	1.07 (0.64–1.81)	0.795	1.91 (1.04–3.52)	**0.037**	1.08 (0.59–1.97)	0.812

^1^; Adjusted for age, gender and sexual orientation; ^2^ *AOR* adjusted odds ratio, *REF* reference category

*p* value for a significant result

### Repeated measures analysis

**Fig. 1:**
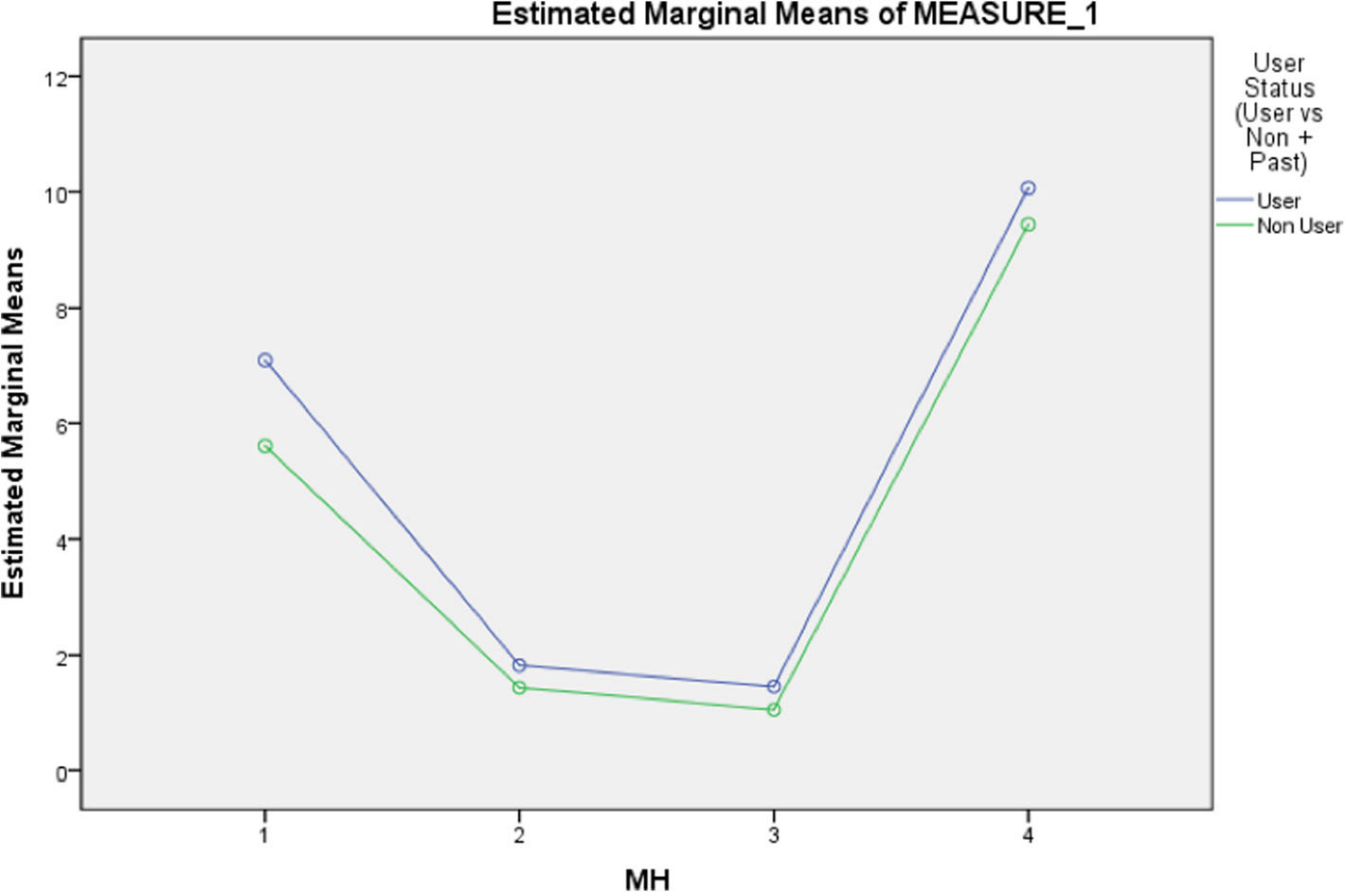
Estimated marginal means of psychological distress (1), anxiety (2), depression (3) and self-esteem (4) by user status

Table 6 displays the relationship between SBDA use and the four mental health scores analysed together adjusted for age and gender. Thus, the repeated measure of mental health consisting of psychological distress, anxiety, depression and self-esteem was the within subject design factor. The mental health by user status interaction was significant (*P* = 0.009, *p* = 0.037) after adjusting for the following: gender*mental health (*p* = 0.001, *p* = 0.005) and age*mental health (*p* < 0.001). The following interaction effects were found not to be significant: gender*user status and age*user status (results not shown). Figure 1 and Table 7 show that the estimated marginal mean scores are significantly higher for users when compared to non-users for three of the four mental health outcome measures: psychological distress (1), anxiety (2), and depression (3). Self-esteem (4) exhibited a higher marginal mean for users but not significantly, due to larger standard errors. In summary, the primary result of interest is that being a SDBA user was significantly associated with increased mental health scores on three of the four outcome measures after adjusting for age and gender.

**Table 6:** Comparison between current dating app users (*n* = 127) and non-users (*n* = 297) adjusted for age and gender on combined mental health outcome

Effect	Wilks’ Lambda	F	Degrees of freedom^1^	Error degrees of freedom	*P*-value	Greenhouse Geiser *p*- value
Mental health	0.275	365.74	3	417	**< 0.001**	**< 0.001**
Mental health * gender	0.961	5.61	3	417	**0.001**	**0.005**
Mental health * age	0.916	6.21	6	834	**< 0.001**	**< 0.001**
Mental health * user status	0.973	3.89	3	417	**0.009**	**0.037**

*p* value for a significant result

**Table 7:** Marginal means estimates for psychological distress (1), anxiety (2), depression (3) and self-esteem (4) by user status

	User Status	Marginal mean estimates (95% CI)	Standard error	
				*P*-value
Psychological distress	User	7.09 (6.30–7.89)	0.40	**0.001**
	Non-User	5.61 (5.11–6.12)	0.26	
Anxiety	User	1.82 (1.54–2.10)	0.14	**0.015**
	Non-User	1.43 (1.26–1.61)	0.09	
Depression	User	1.45 (1.21–1.70)	0.13	**0.005**
	Non-User	1.05 (0.90–1.21)	0.08	
Self-esteem	User	10.07 (9.10–11.04)	0.49	0.261
	Non-User	9.44 (8.83–10.06)	0.31	

*p* value for a significant result

## Discussion

The repeated measures analyses demonstrated a significant association between SBDA use and higher levels of psychological distress, and symptoms of anxiety and depression, however not low self-esteem. The multivariate logistic models found a significant association with psychological distress and depression, however not with anxiety.

These findings support our hypothesis, in part. We hypothesised that SBDA use would be associated with higher levels of psychological distress, anxiety and depression, which was upheld by our results. However, our hypothesis that low self-esteem would also be associated with SBDA use was not statistically supported by the findings. This is particularly interesting given the findings of Strubel and Ronnenberg’s previous studies [8]. We note that a trend for lower self-esteem was found however this was not statistically significant. On the contrary, Strubel & Petrie found a trend and theirs reached significance [8].

The association of SBDA use with higher scores of anxiety and depression symptoms may reflect a causative process; however, we cannot conclude this based on this cross-sectional study. This association may be mediated by the validation-seeking behaviour that has been found to be a motivating factor in SBDA use [8, 24]. Alternatively, it may be that individuals with higher psychological distress, anxiety and depression are more likely to use SBDAs; this could be due to the lower social pressures of these interactions compared to initiating romantic connections face-to-face.

Individuals who used SBDAs daily and those who had used them for more than a year were both found to have statistically significantly higher rates of psychological distress and depression; this is a similar trend to that found with greater duration and frequency of social media use [15, 23]. These findings suggest that the impact of SBDA use on users’ mental health and wellbeing may be dose-dependent. It also suggests that patterns of this impact may parallel those of social media use in other ways, for instance being more pronounced with greater validation-seeking and social comparison [22, 23], or with problematic patterns of use [20, 21]; this is an important area for future research.

### Strengths & Limitations

Limitations of this study include the use of self-reporting, convenience sampling and selection bias. Another limitation of the study is that the mental health outcome measures were categorised which leads to loss of data. While the use of validated brief tools to measure mental health outcomes is a strength, the tools selected potentially limited their accuracy when compared to the more elaborate versions. Considering the inconvenience and potential reluctance towards survey completion, the authors determined that shorter measures would facilitate higher response rates by avoiding survey fatigue and thus render more meaningful data.

The large sample size of the study (*n* = 437) is a strength, however the sample was not representative of the total population due to selection bias and potentially over-representing individuals with a particular interest in dating applications and mental health. Furthermore, the sample was 58.4% (253/433) female and 13.3% (58/437) LGBTQI+ individuals, compared to 50.7 and 3.2% of the Australian population, respectively [32]. Australian women [33, 34] and LGBTQI+ individuals [35] experience greater levels of psychological distress, and have higher rates of anxiety and depression, when compared to men and heterosexual individuals, respectively. This was reflected in our results as women and LGBTQI+ individuals had higher levels of anxiety, depression and low self-esteem, and indicates that our sample may have overrepresented individuals already predisposed to higher rates of adverse mental health than the general Australian population.

Furthermore, the cross-sectional design of the study precludes us from drawing any causative conclusions. However, as a preliminary study in an area with a current paucity of research [27–29, 31], this study has demonstrated an association between SBDA use and poorer mental health outcomes. Future research is recommended to investigate the strength and accuracy of this association using longer forms of validated tools, in a representative sample, and over multiple time points to assess the direction of causality. We also recommend that other factors may need to be considered in future research including participants’ previous physical or mental health and historical relationship patterns.

#### Clinical implications & future directions

Our findings contribute to understanding the impact SBDAs have on psychological distress, anxiety, depression, and self-esteem, keeping the limitations in mind. App developers could potentially reach out to their audience with messages to maintain positive mental health. While causality cannot be ascertained, these results may reflect that SBDA users are an at-risk population, and that the association warrants further investigation. Further research into the effects and mediators of effects of SBDA use on the mental health and psychological wellbeing of users is warranted, particularly regarding the role of motivation and validation-seeking in SBDA use.

## Conclusion

Current SBDA users were found to have significantly higher rates of psychological distress, anxiety and depression, but were not found to have significantly lower self-esteem. The limitations of this study were the cross-sectional study design, a non-representative sample and reliance on self-reporting. SBDA developers can potentially use this information to maintain positive mental health with their users. Future research examining the impact of specific patterns of SBDA use on mental health (such as the impact of multiple SBDA use) would help identify factors of SBDA use that influence mental health.

## Data Availability

The datasets generated and/or analysed during the current study are not publicly available due the data being used for specified purposes within the ethics approval.

## Abbreviations

AOR: Adjusted odds ratio
GAD-2: Generalised Anxiety Disorder-2 scale
K6: Kessler Psychological Distress Scale
MH: Mental Health
OR: Odds ratio
PHQ-2: Patient Health Questionnaire-2
REF: Reference category
RM ANOVA: Repeated Measures Analysis Of Variance
RSES: Rosenberg Self-Esteem Scale
SBDA: Swipe Based Dating Applications

## References

[1] Iqbal M. Tinder Revenue and Usage Statistics (2018). Business of Apps [Internet]. 2019 Feb 27 [cited 2019 Apr 13]. Available from: http://www.businessofapps.com/data/tinder-statistics/.

[2] Giuliano K. Tinder swipes right on monetization. CNBC [Internet]. 2015 Mar 2 [cited 2019 Apr 14]. Available from: https://www.cnbc.com/2015/03/02/-monetization.html.

[3] Murnane K. Report Shows More People Of All Ages Are Dating Online. Forbes [Internet]. 2018 Mar 2 [cited 2019 Apr 14]. Available from: https://www.forbes.com/sites/kevinmurnane/2016/03/02/pew-report-who-uses-mobile-dating-apps-and-online-dating-sites/#3410f52f66e3.

[4] Clement J. Online dating in the United States - Statistics & Facts United States: Statista; 2019 [Available from: https://www.statista.com/topics/2158/online-dating/.

[5] Only 1 in 3 US Marriage Proposals Are a Surprise; Engagement Ring Spend Rises, According to The Knot 2017 Jewelry & Engagement study [press release]. 9 Nov 2017.

[6] eHarmonyUK. The Future of Dating: A study of trends in relationship formation in the UK 1996-2040. London: eHarmony UK; 2014.

[7] Sawyer AN, Smith ER, Benotsch EG (2018). Dating application use and sexual risk behavior among young adults. Sex Res Soc Policy.

[8] Strubel J, Petrie TA (2017). Love me tinder: body image and psychosocial functioning among men and women. Body Image..

[9] Smith A. 15% of American adults have used online dating sites or Mobile dating apps [Internet]. Washington DC: Pew Research Centre; 2016 Feb 11 [cited 2019 Apr 10]. Available from: https://www.pewinternet.org/2016/02/11/15-percent-of-american-adults-have-used-online-dating-sites-or-mobile-dating-apps/.

[10] Australialn Institute of Health and Welfare. Mental Health Services - In Brief 2018 [Internet]. Canberra: AIHW; 2018 [cited 2019 Apr 4]. 48 p. Cat. no. HSE 211. Available from: https://www.aihw.gov.au/getmedia/0e102c2f-694b-4949-84fb-e5db1c941a58/aihw-hse-211.pdf.aspx?inline=true.

[11] Australian Bureau of Statistics. National Survey of Mental Health and Wellbeing: Summary of Results [Internet]. Canberra: ABS; 2007 [cited 2019 Feb 14]. Available from: http://www.ausstats.abs.gov.au/ausstats/subscriber.nsf/0/6AE6DA447F985FC2CA2574EA00122BD6/$File/National%20Survey%20of%20Mental%20Health%20and%20Wellbeing%20Summary%20of%20Results.pdf.

[12] Galderisi SH, Heinz A, Kastrup M, Beezhold J, Sartorius N (2015). Toward a new definition of mental health. World Psychiatry.

[13] Rönnestad M. Swiping right for love? A study about the relationship between tinder usage and self-esteem. Uppsala University’s Publications. 2018.

[14] Tran A SC, Mattie H, Davison K, Agénor M, Austin SB. Dating app use and unhealthy weight control behaviors among a sample of U.S. adults: a cross-sectional study. J Eating Disorders. 2019;7(1):16. 10.1186/s40337-019-0244-4. 10.1186/s40337-019-0244-4 PMC6543621 31164984

[15] Lin LY, Sidani JE, Shensa A, Radovic A, Miller E, Colditz JB (2016). Association between social media use and depression among U.S. young adults. Depression Anxiety.

[16] Primack BA, Shensa A, Escobar-Viera CG, Barrett EL, Sidani JE, Colditz JB (2017). Use of multiple social media platforms and symptoms of depression and anxiety: a nationally-representative study among U.S. young adults. Comput Hum Behav.

[17] Berryman C, Ferguson CJ, Negy C (2018). Social media use and mental health among young adults. Psychiatric Quarterly.

[18] Banjanin N, Banjanin N, Dimitrijevic I, Pantic I (2015). Relationship between internet use and depression: focus on physiological mood oscillations, social networking and online addictive behavior. Comput Hum Behav.

[19] Davila J, Hershenberg R, Feinstein BA, Gorman K, Bhatia V, Starr LR (2012). Frequency and quality of social networking among young adults: associations with depressive symptoms, rumination, and corumination. Psychol Pop Media Cult.

[20] Marino C, Gini G, Vieno A, Spada MM (2018). The associations between problematic Facebook use, psychological distress and well-being among adolescents and young adults: a systematic review and meta-analysis. J Affect Disord.

[21] Shensa A, Escobar-Viera CG, Sidani JE, Bowman ND, Marshal MP, Primack BA (2017). Problematic social media use and depressive symptoms among U.S. young adults: A nationally-representative study. Soc Acience Med.

[22] Stapleton PL, Luiz G, Chatwin H (2017). Generation validation: the role of social comparison in use of Instagram among emerging adults. Cyberpsychol Behav Soc Netw.

[23] Yoon SKM, Mertz J, Brannick M (2019). Is social network site usage related to depression? A meta-analysis of Facebook–depression relations. J Affecive Disorders.

[24] Sumter SR, Vandenbosch L, Ligtenberg L (2017). Love me tinder: untangling emerging adults’ motivations for using the dating application tinder. Telematics Inform.

[25] Miller B (2015). “Dude, Where’s your face?” self-presentation, self-description, and partner preferences on a social networking application for men who have sex with men: a content analysis. Sexuality Culture.

[26] Moradi B (2010). Addressing gender and cultural diversity in body image: objectification theory as a framework for integrating theories and grounding research. Sex Roles.

[27] Plummer F, Manea L, Trepel D, McMillan D (2016). Screening for anxiety disorders with the GAD-7 and GAD-2: a systematic review and diagnostic metaanalysis. Gen Hosp Psychiatry.

[28] Kessler RC, Green JG, Gruber MJ, Sampson NA, Bromet E, Cuitan M (2010). Screening for serious mental illness in the general population with the K6 screening scale: results from the WHO world mental health (WMH) survey initiative. Int J Methods Psychiatr Res.

[29] Lowe B, Wahl I, Rose M, Spitzer C, Glaesmer H, Wingenfeld K (2010). A 4-item measure of depression and anxiety: validation and standardization of the patient health Questionnaire-4 (PHQ-4) in the general population. J Affect Disord.

[30] Carey M, Boyes A, Noble N, Waller A, Inder K (2016). Validation of the PHQ-2 against the PHQ-9 for detecting depression in a large sample of Australian general practice patients. Aust J Prim Health.

[31] Robins RW, Hendin HM, Trzesniewski KH (2001). Measuring global self-esteem: construct validation of a single-item measure and the Rosenberg self-esteem scale. Personal Soc Psychol Bull.

[32] Wilson T, Shalley F. Estimates of Australia’s non-heterosexual population. Australian Population Studies; Vol 2 No 1 (2018). 2018.

[33] Australian Bureau of Statistics. Psychological Distress [Internet]. Canberra: ABS; 2016 [cited 2019 Feb 23]. Available from: https://www.abs.gov.au/ausstats/abs@.nsf/Lookup/by%20Subject/4364.0.55.001~2014-15~Main%20Features~Psychological%20distress~16.

[34] Australian Bureau of Statistics. Mental and Behavioural Conditions [Internet]. Canberra: ABS; 2016 [cited 2019 Feb 23]. Available from: https://www.abs.gov.au/ausstats/abs@.nsf/Lookup/by%20Subject/4364.0.55.001~2014-15~Main%20Features~Mental%20and%20behavioural%20conditions~32.

[35] National LGBTI Health Alliance. The Statistics At A Glance: The Mental Health Of Lesbian, Gay, Bisexual, Transgender And Intersex People In Australia [Internet]. Sydney: National LGBTI Health Alliance; 2016 [cited 2019 Apr 24]. Available from: https://lgbtihealth.org.au/statistics/.

